# Obesity related eating behaviour patterns in Swedish preschool children and association with age, gender, relative weight and parental weight - factorial validation of the Children's Eating Behaviour Questionnaire

**DOI:** 10.1186/1479-5868-8-134

**Published:** 2011-12-08

**Authors:** Viktoria Svensson, Linda Lundborg, Yingting Cao, Paulina Nowicka, Claude Marcus, Tanja Sobko

**Affiliations:** 1 Department of Clinical Science, Intervention and Technology, Karolinska Institutet, Karolinska University Hospital Huddinge, SE 141 86 Stockholm, Sweden; 2 Department of Biosciences and Nutrition, Karolinska Institutet, Novum, SE 141 83 Huddinge, Sweden

**Keywords:** Eating behaviour, CEBQ, children, obesity, factorial validation.

## Abstract

**Background:**

The Children's Eating Behaviour Questionnaire (CEBQ) is a multi-dimensional, parent-reported questionnaire measuring children's eating behaviours related to obesity risk, i.e. 'enjoyment of food', 'food responsiveness', 'slowness in eating' and 'satiety responsiveness'. It has not previously been validated in a Swedish population, neither on children under the age of 2 years. In the present study we examined the factor structure and the reliability of the Swedish version of the CEBQ, for use in an obesity intervention programme targeting preschool children 1-6 years. Further, the associations between eating behaviours and children's age, gender and relative weight (BMI SDS) and parental weight were investigated.

**Methods:**

Parents to 174 children aged 1-6 years (50% girls, mean age 3.8 years), recruited from five kindergartens in Stockholm, completed the Swedish version of the CEBQ. Data on children's weight and height, parental weight, height and educational level was collected. Children's relative weight was calculated for a subpopulation (mean BMI SDS -0.4, n = 47). Factorial validation (Principal Component Analysis) on all CEBQ items was performed. Differences in eating behaviours by age, gender and parental weight were examined. Correlations between eating behaviours and the child's BMI SDS were analysed controlling for age, gender, parental weight and education in linear regression analyses.

**Results:**

The factor analysis revealed a seven factor solution with good psychometric properties, similar to the original structure. The behaviour scales 'overeating'/'food responsiveness', 'enjoyment of food' and 'emotional undereating' decreased with age and 'food fussiness' increased with age. Eating behaviours did not differ between girls and boys. The children's relative weight was not related to any of the eating behaviours when controlling for age, gender, parental weight and education, and only associated with parental weight status.

**Conclusions:**

Our results support the use of the CEBQ as a psychometric instrument for assessing children's eating behaviours in Swedish children aged 1-6 years. Measuring obesity related eating behaviours in longitudinal and interventional studies would offer opportunities for studying causal effects of eating behaviours in the development of obesity in children.

## Background

One of the strongest risk factors for childhood obesity is parental overweight and obesity [1,2]. To prevent a further increase of obesity in children, there is an urgent need for evidence-based interventions, targeting families in different risk groups [3,4]. Previous studies have suggested that weight problems in children can, to some extent, be explained by individual differences in eating style [5,6]. It would be important to early identify behavioural eating traits that promote overeating and obesity in order to address this in multifaceted interventions directed to parents [7].

Eating behaviour patterns develop already in infancy, as children's genetic predispositions, natural food responses and taste preferences are influenced by the exposure to foods and parental feeding practices [8]. The regulation of energy intake differs between children already in the preschool period, and the individual differences in self-regulation and eating behaviours have been associated with heritability, differences in child-feeding practices and with children's adiposity [8-11].

Specific eating behaviours that have been associated with obesity include under-responsiveness to internal satiety cues (low satiety responsiveness, high speed of eating) and over-responsiveness to external food cues such as taste, smell, availability and emotions (high enjoyment of food, food responsiveness and emotional overeating) [5,12], whereas the findings regarding the relationships between fussy eating and relative weight in children have been conflicting [9,13,14].

Several psychometric instruments have been developed for the purpose of detecting individual differences in eating behaviours in children, such as the Dutch Eating Behaviour Questionnaire (DEBQ) and the Children's Eating Behaviour Inventory [15,16]. One of the most comprehensive is the Children's Eating Behaviour Questionnaire, CEBQ, developed and validated in the UK and applicable to preschool children [17,18]. The CEBQ is a multi-dimensional, parent-reported questionnaire measuring children's eating behaviour related to obesity risk. The CEBQ consists of 35 items originally derived from interviews with parents about their children's eating behaviour and the literature on the subject. The items cover eight dimensions of eating style: 'enjoyment of food' (EF), 'food responsiveness' (FR), 'emotional overeating' (EOE), 'desire to drink' (DD), 'satiety responsiveness' (SR), 'slowness in eating' (SE), 'emotional undereating' (EUE) and 'food fussiness' (FF). The CEBQ has been shown to have good internal consistency, adequate test-retest reliability and construct validity [17,18].

The CEBQ subscales are categorized in 'food approach' (EF, FR, EOE, DD) and 'food avoidant' sub-scales (SR, SE, EUE, FF), where the 'food approach' behaviours have been positively associated and the 'food avoidant' scales negatively associated with children's relative weight [17,19-22]. The 'enjoyment of food' scale represents a general interest in food and the 'food responsiveness' scale is intended to measure eating in response to external food cues. These behaviours have been seen to become more apparent as children get older [18,23]. The scale 'desire to drink' was developed in order to detect increased desire to have drinks, particularly sugar-sweetened drinks and has been related to a liking for consuming sweetened drinks [18,24]. 'Satiety responsiveness' reflects the ability to regulate the amount of food that is eaten according to internal satiety cues. This ability has been seen to be high in infants and to decrease with age [18,19,22,23]. The scale 'slowness in eating' measures the speed of eating during the course of a meal and reflects a gradually reduced interest in a meal. This behaviour has been seen to decrease with age, that is older children eat faster than younger children [18,23]. 'Food fussiness' reflects a lack of interest in food and unwillingness to try new foods (food neophobia), leading to an inadequate variety of foods [25], and generally this behaviour decreases with age after the preschool period [23,26]. Finally, the scales 'emotional overeating' and 'emotional undereating' are characterized by either increased or decreased eating in response to negative emotions, such as anger and anxiety.

CEBQ has been successfully used in several child populations to measure associations between eating behaviours and children's relative weight (BMI SDS or BMI z-score): in the UK (age 3-8 years), the Netherlands (age 6-7 years), Portugal (age 3-13 years) and Canada (age 4-5 years) [18-20,22]. The instrument has also been used in other studies, e.g. to compare appetite preferences in children of lean and obese parents, to study continuity and stability in children's eating behaviour during childhood and relationships between temperament and eating behaviours in young children [23,27,28]. The results of these studies support the use of CEBQ in obesity related research.

Eating behaviours of young children are addressed in the Swedish Early STOPP obesity prevention intervention, targeting preschool children 1-6 years of age at high risk of developing obesity [29]. The CEBQ questionnaire is one of several instruments measuring the effectiveness of the Early STOPP intervention, a 5 year intervention starting when the children are 1 years old. However, the CEBQ questionnaire has not previously been validated in a Swedish population; neither has it been validated for children under the age of 2. The primary aim of this study was therefore to examine the factor structure and the reliability of the Swedish version of the CEBQ questionnaire in a population of preschool children corresponding to a cross-section of the Early STOPP study population. The secondary aim was to examine the associations between eating behaviours and child age, gender and relative weight and parental weight and to specifically explore the effect of including children under 2 years of age on the CEBQ factor structure and its variation with age.

## Methods

### Procedure

The study was approved by the Stockholm regional ethics committee. The original English version of the CEBQ had previously been translated into Swedish by another research team (Nowicka and Flodmark from the Skåne University Hospital) using authorized translators. The translation process included two translators who independently translated the questionnaire from English into Swedish. The two translations were compared and the differences were discussed with each translator. Secondly, the same two translators performed the back-translation into English. Comparisons with the original version were performed. Any differences were discussed with the translators and finally with the research team.

The study was performed in parallel with the initiation of the Early STOPP intervention, on a separate population of children with ages corresponding to a cross-section of the Early STOPP population. Families with children 1-6 years old were recruited from five kindergartens within two areas of Stockholm. The CEBQ was distributed together with a cover letter and additional questions regarding the child's birth date, gender, chronic disease, the most current weight and height record from the child healthcare centre and both parents' weight, height, educational level (elementary school, high school, college/university), ethnicity and which of the parents that responded to the questionnaire. The parents filled out the questionnaire at home and mailed it back to the research team.

### Study sample

Parents of 193 children completed and returned the questionnaire (46% response rate; the questionnaire was handed out to 418 families). Children with a chronic disease with potential impact on eating behaviours (such as diabetes, hypothyroidism, acid refluxe disease, bronchopulmonary dysplasia, lactose intolerance) were excluded (n = 10) as well as children with missing age (n = 1) and children with missing parental data (n = 8). The resulting study sample constituted 174 children and their parents. Only if measured within two months prior to answering the CEBQ questionnaire, the weight and height records from the healthcare centre were used, resulting in a sub-population of 47 children with accurate weight and height data. The mean age of the children was 3.8 (SD 1.4) years, 50% of the children were girls. The mother completed the questionnaire for 76% of the children, the father for 20% and both parents in 5% of the cases. A large proportion of the parents had a college/university degree, 72% of the mothers and 69% of the fathers in the whole study sample and 64% of the mothers and 55% of the fathers in the sub-population. In 24% of the families one or both parents had a foreign background (Table 1).

**Table 1:** Anthropometric and demographic characteristics of the families.

	Total population, n = 174	**Sub-population, n = 47 **^***a)***^
	*Mean (SD), min-max*	*Mean (SD)*

*Children's age (years)*	3.8 (1.4), 1.0-6.3	

*Children's BMI SDS*^* b)*^		-0.41 (1.09)

*Parental BMI (kg/m^2^)*		

Mother	22.6 (3.4), 17.7-37.2	

Father	24.9 (2.5), 18.3-33.1	

	*N (%)*	*N (%)*

*Children's sex*		

Girls	87 (50.0)	27 (57.4)

Boys	87 (50.0)	20 (42.6)

*Children's age groups*		

1 year	25 (14.4)	

2 years	30 (17.2)	

3 years	34 (19.5)	

4 years	39 (22.4)	

5-6 years	46 (26.4)	

*Parental weight categories *^c)^		

Mother		

Normal weigth (BMI < 25)	149 (85.6)	

Overweight (BMI 25-29.9)	19 (10.9)	

Obese (BMI ≥30)	6 (3.4)	

Father		

Normal weigth (BMI < 25)	104 (59.8)	

Overweight (BMI 25-29.9)	64 (36.8)	

Obese (BMI ≥ 30)	6 (3.4)	

Mother and father combined weight groups		

2 overweight or at least 1 obese parent	20 (11.5)	10 (21.3)

2 normal weight or 1 normal weight and 1 overweight parent	154 (88.5)	37 (78.7)

*Parental education*		

Mother		

Elementary school	4 (2.3)	1 (2.1)

High school	44 (25.3)	16 (34.0)

College/University	126 (72.4)	30 (63.8)

Father		

Elementary school	2 (1.1)	2 (4.3)

High school	52 (30.0)	19 (40.4)

College/University	120 (69.0)	26 (55.3)

*Parental ethnicity*		

2 parents Swedish	132 (75.9)	

1 or 2 parents non-Swedish	42 (24.1)	

*Completed the questionnaire*		

Mother	132 (75.9)	

Father	34 (20.0)	

Mother and father	8 (4.6)	

^a) ^For the sub-population only the variables used in the analyses between eating behaviours and children's relative weight (BMI SDS) are reported.

^b) ^BMI Standard deviation score, according to a reference population [30]

^c) ^Weight categories according to international cut-off points [31]

### Data definitions and processing

Body mass index, BMI, was calculated as weight/height^2 ^for children and parents. BMI SDS (standard deviation score), a measure of relative weight in children that is gender and age independent, was calculated for children with accurate weight and height measures using a reference standard [30]. International cut off points were used to classify children from 2 years in weight categories (normal weight, overweight and obese [31]. Based on parental BMI each parent was categorized as normal weight (BMI < 25), overweight (BMI 25-29.9) or obese (BMI ≥ 30). The children were divided in two groups based on the combination of the parental weight categories: children with at least one obese parent or two overweight parents versus children with two normal weight parents or one normal weight and one overweight parent. The children were also subdivided into five approximately equally-sized age groups: 1 year, 2 years, 3 years, 4 years and 5-6 years old. The mean BMI SDS of the subpopulation was -0.4 (SD 1.1), Table 1. All children had normal BMI for their age.

Each item of the CEBQ was answered using a five-point Likert frequency scale (1 = never, 2 = rarely, 3 = sometimes, 4 = often, 5 = always). Missing CEBQ data was handled using the median substitution method (in total 7 missing responses, no item and no individual had several missing responses). For five of the items the scores were reversed, due to opposite phrasing, according to instrument instructions.

### Statistical analyses

Factor analysis was performed to analyse the underlying structure of the Swedish version of questionnaire and determine whether the structure was similar to the original CEBQ. Principal Components Analysis (PCA) with Varimax normalized rotation as well as with Direct oblimin rotation was run on all 35 items of the CEBQ. The number of factors was set to eight since the original questionnaire had an eight-factor structure. The PCA with Varimax normalized rotation was performed both on the whole population and in addition on a subpopulation excluding children under 2 years of age (n = 25). Different thresholds for factor loadings were tested, 0.40, 0.50 and 0.60. Internal reliability coefficients (Cronbach's alpha) was determined and used in optimizing the factor structure. Descriptive statistics for the scales as defined through the PCA were calculated (mean, SD). A higher mean score indicated a higher presence of the eating behaviour. Pearson's correlations between factors were determined.

Differences in the children's eating behaviours by gender and parental weight groups were tested using independent sample t-tests. Variation in age was analysed using analysis of variance (one-way ANOVA) with post-hoc polynomial contrast tests examining significant linear trends. Linear correlations (Pearson's correlation coefficient) were examined between each subscale of CEBQ and paternal BMI, maternal BMI and child BMI SDS. Finally, multivariate linear regression analysis was used to analyse the association between the children's BMI SDS (dependent variable) and each of the eating behaviour scales, controlling for child age and gender, parental weight group and parental educational level (elementary school was used as a reference category). P-values below 0.05 were regarded as statistical significant. STATISTICA (data analysis software system, version 10. StatSoft, Inc. http://www.statsoft.com) was used to perform all the statistical analyses, with the exception for the PCA with Direct oblimin rotation where SPSS Statistics 18.0 (SPSS Inc., Chicago, IL, USA) was used.

## Results

### Factor analysis

The factor analyses revealed a seven-factor solution, which accounted for 61.2% of the total variance (Table 2). The two PCA analyses on the full sample resulted in the same factor structure (the result of the PCA with Varimax normalized rotation is reported). The factor analysis on the subpopulation where 1 year old children were excluded confirmed the structure. Items from two of the 'food-approach' scales, FR and EOE, loaded onto the same factor (called 'overeating', EOE/FR). One item from the original FR scale loaded onto a separate eighth scale, but this factor was disregarded, even though the loading was high, since the other FR items (4 of 5) loaded onto the combined EOE/FR scale. Most of the items loaded as expected and the factor loadings were comparable to the original study and other validation studies [18,19,22]. Only three items loaded differently compared to the original study. 'My child has a big appetite' had the highest loading on the EF scale and not on the SR scale to which it originally belonged, and was therefore retained on the EF scale (with reversed scores). The item 'My child is always asking for food' originally belonging to the FR scale loaded onto two factors, DD and FR, with the same magnitude of loading (0.45 and 0.41 respectively). This item was retained on the EOE/FR scale on a theoretical basis. The third item that did not load as expected was the above mentioned FR item that loaded onto a separate scale, 'Even if my child is full up s/he finds room to eat his/her favourite food'. One item with a factor loading below 0.40 was excluded in further analyses, 'My child eats more and more slowly during the course of a meal', optimizing the internal reliability of the SE scale.

**Table 2:** Factor loadings of the factor analysis (Principal Components analysis with Variamax normalized rotation) with all 35 items of CEBQ.

Scale name and items	Loading	Scale name and items	Loading
**Food fussiness FF (Factor 1; 12.4% variance)**		**Desire to drink DD (Factor 5; 7.5% variance)**	

My child refuses new food at first	0.85	My child is always asking for a drink	0.77

My child enjoys tasting new foods	0.86	If given the chance, my child would drink continuously throughout the day	0.81

My child enjoys a wide range of foods	0.65	If given the chance, my child would always be having a drink	0.83

My child is difficult to please with meals	0.57		

My child is interested in tasting food s/he hasn't tasted before	0.87	**Satiety responsiveness SR (Factor 8; 6.9% variance)**	

My child decides that s/he doesn't like a food, even without tasting it	0.77	*My child has a big appetite^e)^*	*0.32*

		My child leaves food on his/her plate at the end of a meal	0.65

**Emotional overeating/Food responsiveness EOE/FR (Factor 2; 9.2% variance)**^**a)**^		My child gets full before his/her meal is finished	0.76

My child eats more when worried (EOE)	0.72	My child gets full up easily	0.62

My child eats more when annoyed (EOE)	0.73	My child cannot eat a meal if s/he has had a snack just before	0.60

My child eats more when anxious (EOE)	0.49		

My child eats more when s/he has nothing else to do (EOE)	0.75	**Slowness in eating SE (Factor 6; 6.1% variance)**	

My child is always asking for food (FR)^b)^	0.41	My child finishes his/her meal quickly	0.80

If allowed to, my child would eat too much (FR)	0.67	My child eats slowly	0.86

Given the choice, my child would eat most of the time (FR)^c)^	0.54	My child takes more than 30 minutes to finish a meal	0.59

If given the chance, my child would always have food in his/her mouth (FR)^d)^	0.48	*My child eats more and more slowly during the course of a meal^f)^*	*0.30*

*Even if my child is full up s/he finds room to eat his/her favourite food (FR)^g)^*	*0.03*		

		***Food responsiveness FR (Factor 7; 5.0% variance)^g)^***	

**Emotional under-eating EUE (Factor 3; 8.1% variance)**		*Even if my child is full up s/he finds room to eat his/her favourite food^g)^*	*0.63*

My child eats less when angry	0.87	*My child is always asking for food^b) ^*	*0.18 *

My child eats less when s/he is tired	0.72	*If allowed to, my child would eat too much *	*0.33 *

My child eats more when s/he is happy	0.68	*Given the choice, my child would eat most of the time^c)^*	*0.43 *

My child eats less when upset	0.87	*If given the chance, my child would always have food in his/her mouth^d)^*	*0.51 *

			

**Enjoyment of food EF (Factor 4; 11.0% variance)**			

My child loves food	0.73		

My child has a big appetite (SR)^e)^	0.62		

My child is interested in food	0.70		

My child looks forward to mealtimes	0.81		

My child enjoys eating	0.81		

^a) ^All items of the original EOE scale and 4 of 5 items of the original FR scale loaded onto the same factor; thus these were combined into one scale.

^b) ^The item 'My child is always asking for food' (FR) loaded most highly on the DD scale (0.45), but also loaded onto the EOE/FR scale with a loading with the same magnitude (0.41). Retained on the EOE/FR scale on theoretical basis.

^c) ^The item 'Given the choice, my child would eat most of the time' (FR) loaded most highly on the EOE/FR scale (0.54), but also loaded onto the FR scale with a loading with the same magnitude (0.43). Retained on the EOE/FR scale optimizing the internal reliability (Cronbach's alpha) for the EOE/FR scale.

^d) ^The item 'If given the chance, my child would always have food in his/her mouth' (FR) loaded most highly on the FR scale (0.51), but also loaded onto the EOE/FR scale with a loading above of the same magnitude (0.48). Retained on the EOE/FR scale, optimizing the internal reliability (Cronbach's alpha) for the EOE/FR scale.

^e) ^The item 'My child has a big appetite' originally belonged to SR but loaded most highly onto the EF scale, and was therefore retained on the EF scale. In the table this item is additionally reported under the SR scale to compare with the original structure.

^f) ^The item 'My child eats more and more slowly during the course of a meal' (SE) had a loading below 0.4 and was not included in further analyses.

^g) ^One FR item loaded clearly onto a separate factor; however this factor and item was disregarded since the combined EOE/FR scales was created. The contribution of this factor was not included in the total variance reported. In the table this item is additionally reported under the EOE/FR scale to compare with the original structure.

### Reliability

Internal reliability coefficients (Cronbach's alpha) were calculated for each of the resulting seven factors, including the items as presented above (Table 3, Alt. 1). The coefficients ranged from 0.71-0.90, which are all acceptable and comparable to the previous CEBQ validation studies [18,19,22]. As a comparison, Cronbach's alpha was calculated for the factors according to the original structure, range 0.69-0.90 (Table 3, Alt. 2).

**Table 3:** Internal reliability of the CEBQ.

	**Alt 1. According to our factor analysis**^**a)**^		**Alt 2. According to original factor structure**^**b)**^	
	**Cronbach's alpha**	**Average inter-item corr**.	**Cronbach's alpha**	**Average inter-item corr**.

Overeating (EOE/FR)	0.82	0.37	0.79	0.32

Food responsiveness (FR)	0.75	0.43	0.69	0.34

Emotional overeating (EOE)	0.70	0.41		

Enjoyment of food (EF)	0.89	0.62	0.87	0.64

Desire to drink (DD)	0.80	0.59		

Satiety responsiveness (SR)	0.71	0.39	0.74	0.38

Slowness in eating (SE)	0.75	0.53	0.68	0.38

Emotional undereating (EUE)	0.82	0.55		

Food fussiness (FF)	0.90	0.63		

^a) ^Items are included in each factor as given by the result of our factor analysis (see Table 2)

^b) ^Items are included in each factor according to the original structure.

Results only presented for factors that differ in comparison to alternative 1.

- The item 'Even if my child is full up s/he finds room to eat his/her favourite food' is included in the EOE/FR and FR factors

- The item 'My child has a big appetite' is included in the SR factor and excluded from the EF factor

- The item 'My child eats more and more slowly during the course of a meal' is included in the SE factor

### Correlations between scales

The 'food-approach' (EOE/FR, EF, DD) scales and the 'food-avoidant' scales (SR, SE, EUE, FF) tend to be positively inter-correlated, and negatively correlated between the two groups of scales, similar to the previous studies (Table 4) [18,19,22]. The significant correlations between the eating behaviours were medium to strong.

**Table 4:** Pearson's correlations between the CEBQ subscales, seven-factor solution.

CEBQ scales	EOE/FR	EF	DD	SR	SE	EUE	FF
Overeating (EOE/FR)	-						

Enjoment of food (EF)	0.26 **	-					

Desire to drink (DD)	0.39 ***	-0.09	-				

Satiety responsiveness (SR)	-0.16 *	-0.38 ***	0.07	-			

Slowness in eating (SE)	-0.23 **	-0.49 ***	0.18 *	0.38 ***	-		

Emotional undereating (EUE)	0.07	-0.01	0.17 *	0.33 ***	-0.03	-	

Food fussiness (FF)	-0.02	-0.58 ***	0.14	0.33 ***	0.28 ***	0.12	-

* p < 0.05 ** p < 0.01 *** p < 0.001

Bold area upper-left corner: significant inter-correlations between 'food approach' subscales.

Bold area bottom-right corner: significant inter-correlations between 'food avoidant' subscales.

### Age and gender differences

**Figure 1:**
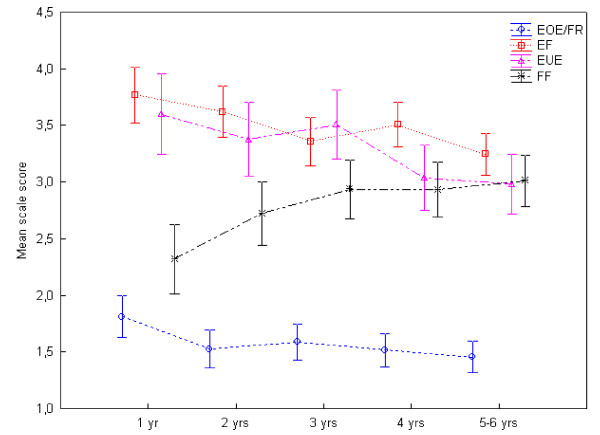
**Mean eating behaviour scores by age groups**. The mean scores of the CEBQ eating behaviours that varied significantly with age: 'overeating' (EOE/FR; p = 0.03), 'enjoyment of food' (EF; p = 0.01) and 'emotional undereating' (EUE; p = 0.01) decreased with age and 'food fussiness' (FF; p = 0.01) increased with age. Vertical bars denote 0.95 confidence intervals.

Girls and boys did not differ in eating behaviours, but variation in age was identified for several of the scales (Table 5, Figure 1). The scores for EOE/FR significantly decreased with age (p = 0.03) and so did the scores for EF (p = 0.01)) and EUE (p = 0.01) whereas the FF scale was found to increase with age (p = 0.01). For all these four scales significant linear trends by age were confirmed by the ANOVA post-hoc polynomial tests (p = 0.01-0.001). The behaviours SR, DD and SE did not vary significantly with age.

**Table 5:** CEBQ subscales, descriptive statistics and differences by gender and age groups, seven-factor solution (n = 174).

	Gender			Age					
	**Boys** **(n = 87)**	**Girls** **(n = 87)**	**p**^**a)**^	**1 year** **(n = 25)**	**2 years** **(n = 30)**	**3 years** **(n = 34)**	**4 years** **(n = 39)**	**5-6 years** **(n = 46)**	**p**^**b)**^
	
	**Mean** **(SD)**	**Mean** **(SD)**		**Mean** **(SD)**	**Mean** **(SD)**	**Mean** **(SD)**	**Mean** **(SD)**	**Mean** **(SD)**	

Overeating (EOE/FR)	1.5 (0.4)	1.6 (0.5)	0.33	**1.8 (0.6)**	**1.5 (0.5)**	**1.6 (0.5)**	**1.5 (0.4)**	**1.5 (0.4)**	0.04 *

Enjoment of food (EF)	3.4 (0.7)	3.5 (0.6)	0.19	**3.8 (0.7)**	**3.6 (0.6)**	**3.4 (0.6)**	**3.5 (0.5)**	**3.2 (0.7)**	0.01 *

Desire to drink (DD)	2.0 (0.8)	2.1 (0.8)	0.45	2.1 (0.8)	2.0 (0.9)	2.1 (0.9)	1.9 (0.6)	2.0 (0.7)	0.76

Satiety responsiveness (SR)	3.1 (0.6)	3.1 (0.6)	0.58	3.1 (0.7)	3.1 (0.6)	3.1 (0.6)	3.0 (0.5)	3.1 (0.6)	0.91

Slowness in eating (SE)	2.7 (0.8)	2.9 (0.7)	0.09	2.4 (0.7)	2.8 (0.6)	2.8 (0.6)	2.7 (0.7)	2.8 (0.8)	0.14

Emotional undereating (EUE)	3.3 (0.9)	3.2 (0.9)	0.23	**3.6 (0.8)**	**3.4 (0.9)**	**3.5 (0.9)**	**3.0 (1.0)**	**3.0 (1.0)**	0.01 *

Food fussiness (FF)	2.9 (0.8)	2.8 (0.8)	0.47	**2.3 (0.7)**	**2.7 (0.8)**	**2.9 (0.7)**	**2.9 (0.7)**	**3.0 (0.9)**	0.01 *

^a) ^P-value from t-test between independent groups.

^b) ^P-value from one-way ANOVA; * p < 0.05

### Correlations between eating behaviours and relative weight

There were no significant correlations between the children's BMI SDS and any of the factors (the magnitude of all correlation coefficients r = 0.01-0.27, n = 47). Significant negative correlations were identified between maternal BMI and EUE (r = -0.21, p = 0.01) and between maternal BMI and FF (r = -0.19, p = 0.01) and a significant positive correlation was found between paternal BMI and the EOE/FR scale (r = 0.16, p = 0.03). The magnitude of all the correlation coefficients between maternal BMI and eating behaviours were 0.01 - 0.21, and between paternal BMI and eating behaviours 0.01 - 0.16 (n = 174). The children's eating behaviours were not significantly different between the parental weight groups (data not shown).

None of the eating behaviour scales was significantly associated with children's BMI SDS, when controlling for age, gender, parental education and parental weight groups in multivariate regression analyses. However, the children's BMI SDS was significantly associated with parental weight groups in all the analyses; the children's BMI SDS was higher in the group with one obese or two overweight parents (p = 0.01-0.02).

## Discussion

This is the first study to use the CEBQ instrument in a preschool sample including children as young as 1 year old, offering unique opportunities to measure eating behaviours very early in life and to analyse their variation with age in the age bracket 1-6 years. We have shown that the Swedish version of the CEBQ has good psychometric properties in terms of factor structure, internal reliability and correlations between subscales, similar to the original UK study and two other validation studies on Dutch and Portuguese samples [18,19,22].

The factor analysis showed that a seven factor structure was the best solution in our sample, combining two of the 'food-approach' scales, 'emotional overeating' and 'food responsiveness' into one ('overeating'), confirming the factor structure of the previous Dutch study [19]. Several items that originally belonged to the 'food responsiveness' scale had loadings above 0.4 on both the 'overeating' and 'food responsiveness' scales, but these were retained on the 'overeating' scale, optimizing the internal reliability. The single item that loaded onto a separate factor was disregarded since one item is not sufficient to describe a dimension of eating behaviour. We chose to use the resulting seven-factor structure in the statistical analyses, rather than the original structure, since it fitted our sample the best and comparisons with other validation studies were still applicable. However, using the original structure would have been an option as the internal reliability was only marginally lower.

Since this study included children as young as 1 year old up to 6 years old, we found age effects for several of the scales that interestingly differed for some of the eating behaviours compared to previous studies. The 'food-approach' behaviours 'overeating' and 'enjoyment of food' was less present in the older preschool children, whereas these scales previously have been seen to increase with age from 2-3 years of age [18,23]. We also observed that 'food fussiness' was more present in older children, whereas this eating behaviour did not vary by age in the original study (study population 3-9 years) and in general is thought to decrease with age after the preschool period [18,23]. As previous studies did not include children as young as 1 year old this may explain our results. Food neophobia, which is described as the reluctance to eat new food, normally starts to develop in children from the age of 18 months, reaches a peak between 2 and 6 years of age and then gradually decreases with age (and with repeated food exposures) [26,32,33]. Food neophobia is measured as part of the CEBQ scale 'food fussiness', which has been shown to be negatively correlated with both 'enjoyment of food' and 'food responsiveness' [18,19]. It would be logical that these eating behaviours have opposite patterns also in variation with age. Since our study did not include school-age children, we suggest that the youngest children might have a lower neophobic behaviour as well as the highest scores for 'enjoyment of food'. A higher presence of 'overeating' in younger children may seem somewhat surprising though, since in theory children's ability to self-regulate how much to eat normally decreases with age [8]. However, the 'overeating' scores were quite low for all children in our sample, and thus the identified difference with regard to age may be of less importance. Similar to previous findings, we could report that the behaviour 'emotional undereating' decreased with age, implying that as children grow older the negative effect of emotions, such as anger and tiredness, on how much the children eat gradually diminishes.

Boys and girls did not differ in eating behaviours, which was comparable with the original study that only saw minimal gender differences [18]. In older (adolescent) children, it has been reported that boys and girls have different eating styles, however it is not known at what age these differences start to develop [34].

Our study could not identify any associations between eating behaviours and the children's relative weight (BMI SDS) - in contrast to previous research [18-20,22]. A plausible explanation is a lack of power, our sample was quite small and weight homogeneous. Other studies analysing associations between relative weight and eating behaviours have had a reasonable share of overweight/obese children in their study samples [19-22,35]. On the other hand we found a significantly higher relative weight among children having one obese or two overweight parents. This result is not surprising since parental weight has been identified as a dominating risk factor for obesity in children and weight is highly heritable [1,2,36,37], but it is in contrast to the previous CEBQ studies [18-20,22]. However, the associations between eating behaviours and the children's relative weight have not been controlled for parental weight in several of the previous studies [19,20,22].

Interestingly, the parents' BMI correlated with certain eating behaviours. Mothers with higher BMI had children with lower scores for the 'food avoidant' scales 'emotional under-eating' and 'food fussiness' and fathers with higher BMI had children showing higher 'overeating' scores. This confirms previous research on the effect of parental weight on children's eating behaviours. In one study, comparing eating behaviours in preschool children to lean and obese parents, children with obese parents showed higher emotional overeating and food responsiveness [28]. The relation between maternal BMI and their sons emotional eating has been seen to be mediated by maternal eating behaviour in a German population of preschool children [38]. There are additional evidence for associations between different aspects of parental behaviour (parents' own eating behaviours, parental feeding practises and parenting style) and children's eating behaviours [13,39]. The identified association between parental and child relative weight could as suggested above partly be explained by genetic factors where one possible pathway is through inherited appetitive traits, which also could explain our associations between parental relative weight and children's eating behaviours [10]. In measuring children's eating behaviours it thus appears to be important to take into account both familial predisposition to obesity as well as parental behavioural influence [7].

This study has some limitations that should be acknowledged. The parents' weight and height were self-reported. Self-reports are known to underestimate BMI, especially among females, but there are epidemiological studies showing that self-reported weight and height in adults has been reliable for recognizing associations [1]. The sample of children for which relative weight was available was small and weight homogeneous, with a negative impact on the possibility to detect associations between eating behaviours and child weight. The external validity may also be limited to a high SES population, due to a large share of parents with high educational level in our sample. Finally, regarding the applicability of the questionnaire for children under 2 years of age, there is a possibility that parents with younger children may have found some items less relevant when describing their child's eating behaviour, with a potential effect on their responses. We chose to use the CEBQ even though it was originally developed and validated on children above 2 years old since we found it to be the most suitable tool available [18]. In addition, approval to use the questionnaire and confirmation that it could be applicable for children under 2 years of age was obtained from the developer of the instrument (prof. Wardle). Our study also confirmed that the factor structure of the CEBQ was unaffected by the youngest children. However, as this study has been completed the authors have become aware that a CEBQ version for toddlers is being developed by the same group (unpublished).

In this study the eating behaviours were assessed among young children only at one occasion. However, the outcome in terms of overweight can only be detected in longitudinal studies, where eating behaviours are measured repeatedly. Eating behaviours would also be an applicable outcome in interventional studies, where parents' knowledge about children's eating behaviours and parents' feeding practices can be targeted. Future research will focus on longitudinal associations between parental feeding behaviour and child eating behaviours in groups with different predisposition to obesity.

## Conclusions

Our study supports the use of the CEBQ as a psychometric instrument for assessing children's eating behaviour in Swedish children aged 1-6 years. The pattern of eating behaviours of children 1 year old seems to differ compared to that of older preschool children. Children's relative weight is not associated with eating behaviours when controlling for parental weight. Measuring obesity related eating behaviours in longitudinal and interventional studies, starting at an early age and in the context of the family environment, offers opportunities for studying causal effects of eating behaviours in the development of obesity in children.

## List of abbreviations used

CEBQ: Children's Eating Behaviour Questionnaire; EF: Enjoyment of food; FR: Food responsiveness; EOE: Emotional overeating; DD: Desire to drink; SR: Satiety responsiveness; SE: Slowness in eating; FF: Food fussiness; PCA: Principal Component Analysis; BMI SDS: Body mass index standard deviation score.

## Competing interests

The authors declare that they have no competing interests.

## Authors' contributions

All authors were involved in all parts of this study and approved the final manuscript. The contributions are listed below.

VS and TS were responsible for the design of the study, obtaining ethics approval as well as overseeing the study implementation, performing the statistical analyses and writing the manuscript. PN was responsible for the translation of the questionnaire, supervising the study design and finalizing the manuscript. LL and YC performed the collection of data, statistical analyses and drafted the manuscript. CM reviewed the study design and finalized the manuscript.
